# Supplementary figures and images for: Intra- and Intertumoral Microglia/Macrophage Infiltration and Their Associated Molecular Signature Is Highly Variable in Canine Oligodendroglioma: A Preliminary Evaluation
Source: Vet Sci. 2023 Jun 19;10(6):403. doi: 10.3390/vetsci10060403 (PMC10303632; doi:10.3390/vetsci10060403)

Beta Actin  
43 kd

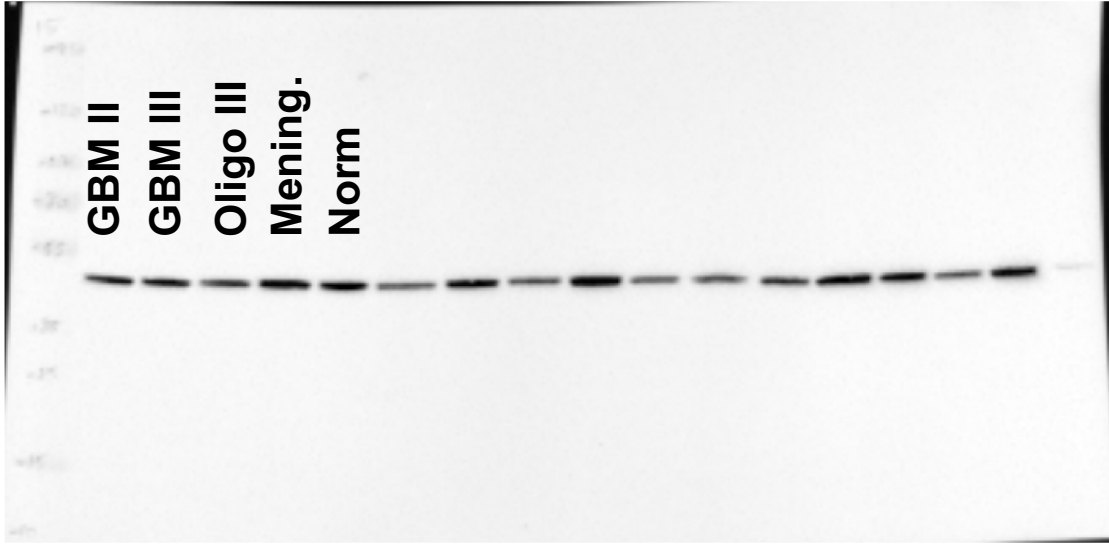

Gal3  
30 kD

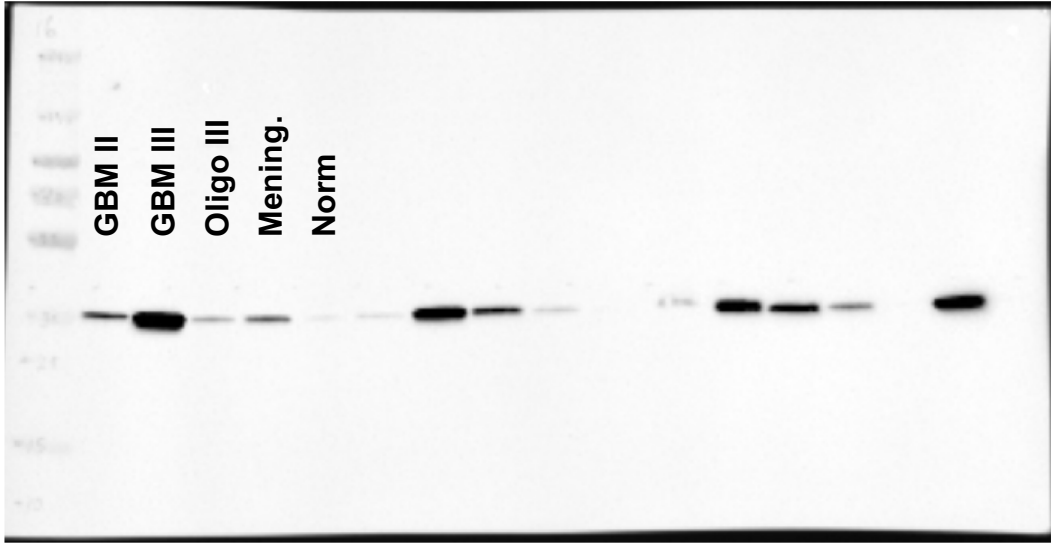

Smad2/3  
58 kd

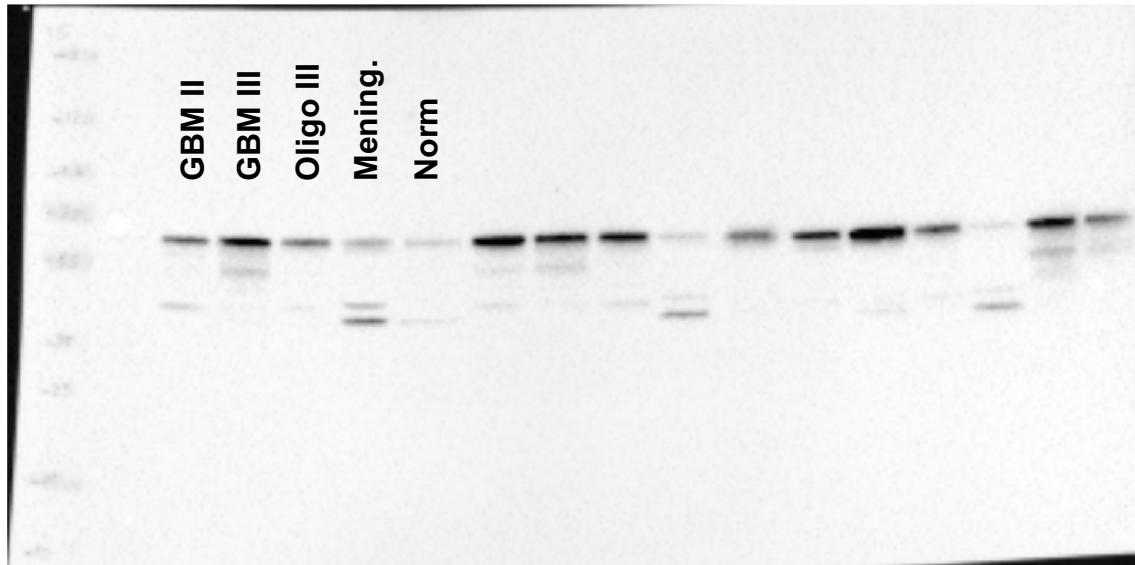

Psmad2/3  
55-60 kD

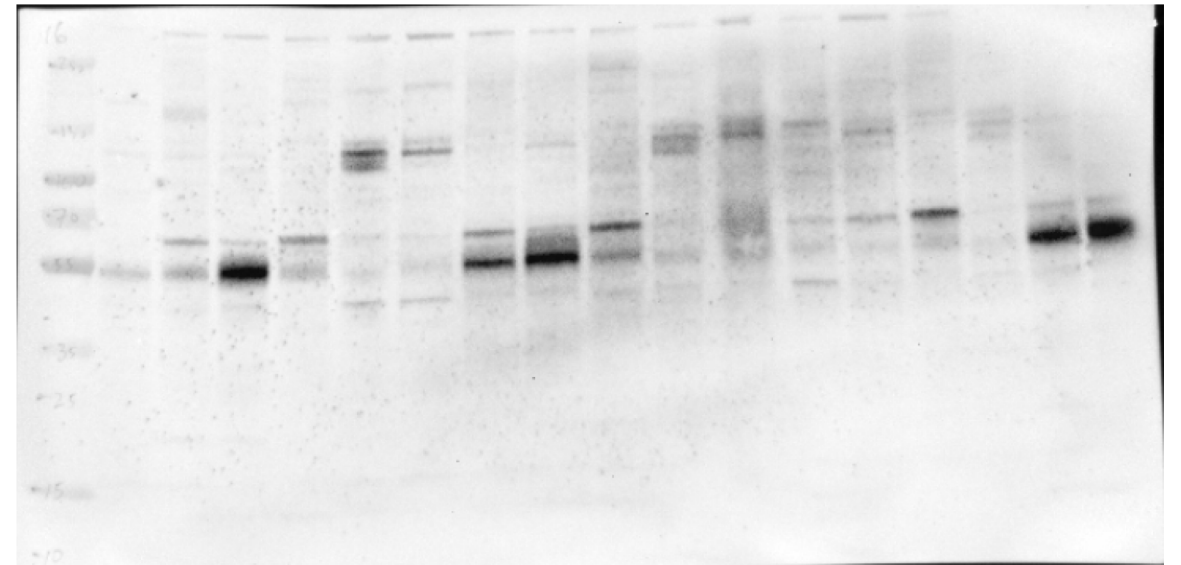

Tgfb1  
44/13

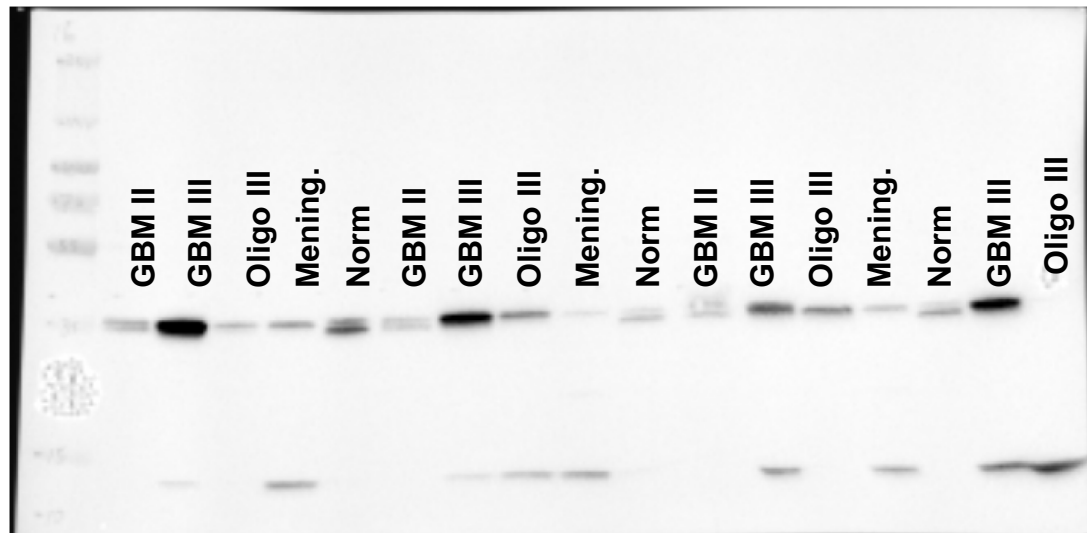

Hsp60  
60kd

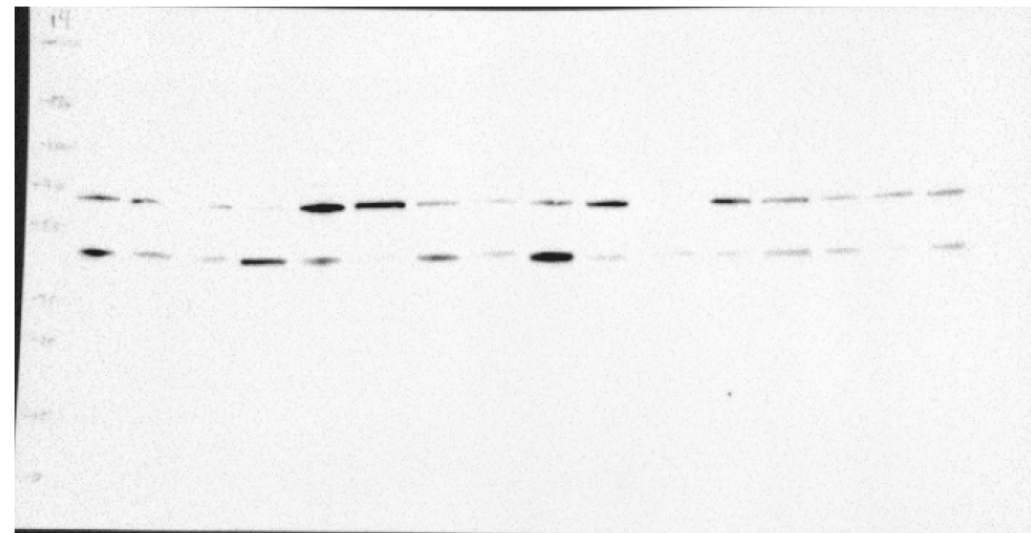

TGFbR1  
53kd

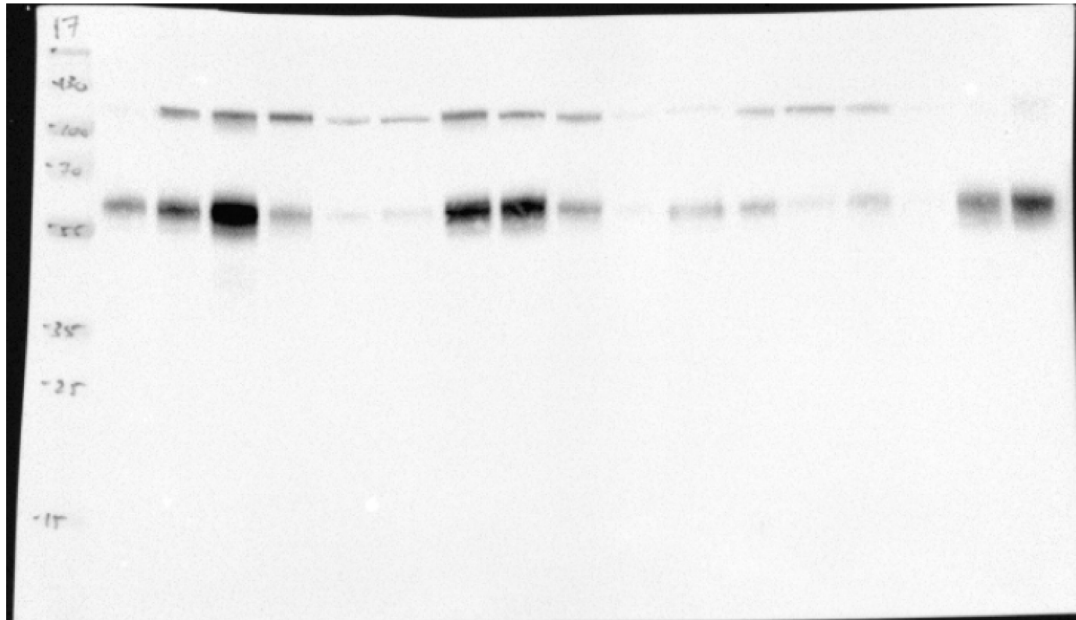

TGFbR2  
64/65/75 kd

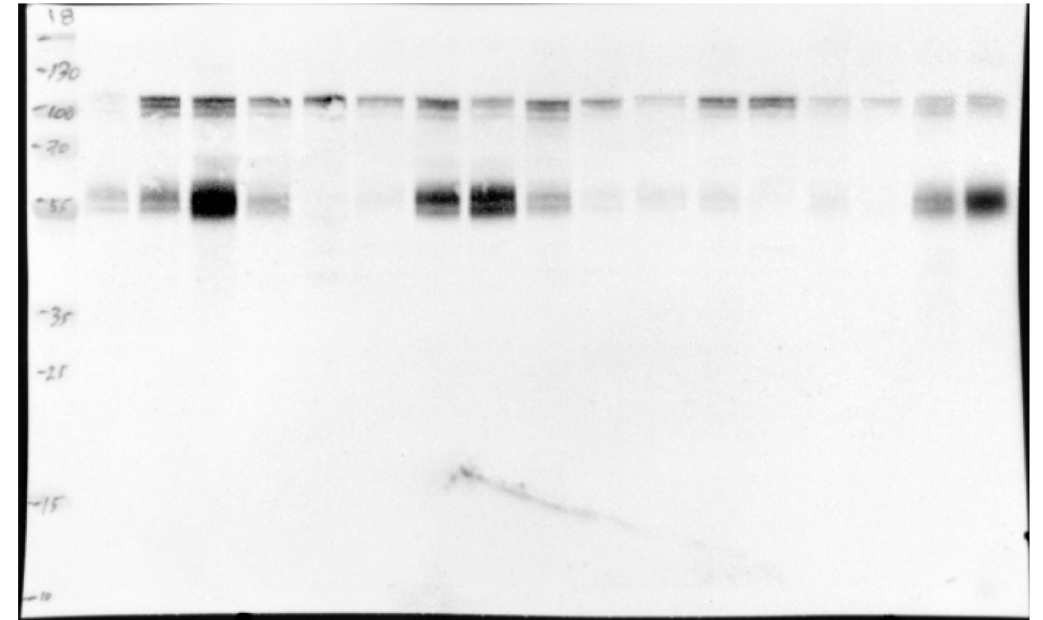

Supplement: Supplementary file 1 [file vetsci-10-00403-s001.zip › vetsci-2442350-supplementary.pdf]
